# Central Administration of Insulin Combined With Resistin Reduces Renal Sympathetic Nerve Activity in Rats Fed a High Fat Diet

**DOI:** 10.3389/fphys.2019.00093

**Published:** 2019-02-11

**Authors:** Hamza Habeeballah, Naif Alsuhaymi, Martin J. Stebbing, Emilio Badoer

**Affiliations:** School of Health and Biomedical Sciences, RMIT University, Melbourne, VIC, Australia

**Keywords:** resistin, insulin, renal sympathetic nerve activity, high fat diet, central interactions, cardiovascular function

## Abstract

Insulin receptors are widely distributed in the central nervous system and their activation by insulin elicits renal sympatho-excitatory effects. Resistin, an adipokine, promotes resistance to the metabolic effects of insulin. Resistin also induces increases in renal sympathetic nerve activity (RSNA) by acting in the brain, but whether it can influence insulin’s actions on RSNA is unknown. In the present study we investigated, in male Sprague-Dawley rats (7–8 weeks of age), the effects of central administration of insulin combined with resistin on RSNA following a normal diet (ND) and a high fat diet (HFD) (22% fat), since HFD can reportedly attenuate insulin’s actions. RSNA, mean arterial pressure (MAP) and heart rate (HR) responses were monitored and recorded before and for 180 min after intracerebroventricular injection of saline (control) (*n* = 5 HFD and ND), resistin (7 μg; *n* = 4 ND, *n* = 5 HFD), insulin (500 mU; *n* = 6 ND, *n* = 5 HFD), and the combination of both resistin and insulin (*n* = 7 ND, *n* = 5 HFD). The key finding of the present study was that when resistin and insulin were combined there was no increase in RSNA induced in rats fed a normal diet or the high fat diet. This contrasted with the sympatho-excitatory RSNA effects of the hormones when each was administered alone in rats fed the ND and the HFD.

## Introduction

Insulin is a hormone produced by the pancreas, in response to elevated blood glucose (Rahmouni et al., 2003). Insulin is released into the circulation and can cross the blood-brain barrier. Insulin receptors are widely distributed in the central nervous system (Havrankova and Roth, 1978; Werther et al., 1987; Marks et al., 1990), and their activation by insulin can influence body weight control, glucose homeostasis and sympathetic nerve activity to the kidney, hindlimb and brown adipose tissues (Woods et al., 1979; Balkan et al., 1993; Kahn et al., 1993; Morgan et al., 1993; Utriainen et al., 1996; Baskin et al., 1999; Rahmouni et al., 2003, 2004; Morgan and Rahmouni, 2010; Cassaglia et al., 2011; Stocker and Gordon, 2015; Spoto et al., 2016).

Insulin resistance is a key underlying component of many pathophysiological conditions such as metabolic syndrome, obesity and Type 2 diabetes. Associated with these metabolic conditions are increased risks of cardiac impairment, vascular disease, kidney and liver disease. Conditions associated with insulin resistance are increasing at an alarming rate and present an ever-increasing health and economic burden worldwide. Insulin resistance can occur in the peripheral tissues as well as the central nervous system (Obici et al., 2002; Morgan and Rahmouni, 2010). The underlying mechanisms that are responsible for insulin resistance include desensitization of the receptor and impaired intracellular signaling processes (Steppan et al., 2005).

Resistin is an adipokine, which is produced by fat cells in rodents and by macrophages that have infiltrated adipose tissue in humans. Although resistin acts systemically, recent studies have now demonstrated that resistin can act in the brain and induces increases in sympathetic nerve activity to the kidney (RSNA) and hindlimb (LSNA) (Kosari et al., 2011, 2012), which is similar to central actions of insulin. Resistin is known to induce resistance to insulin’s peripheral metabolic actions (hence its name) (Muse et al., 2004; Jiang et al., 2016; Santilli et al., 2016), but whether such negative interactions occur in the brain with respect to cardiovascular regulation, and RSNA in particular, is unknown. Such an interaction may be important in conditions in which both hormones are elevated, for example in conditions of high adiposity. Thus, in this study we investigated the effect of combined administration of insulin and resistin on the central control of RSNA.

Consumption of high fat diets (HFD) is known to reduce the effects of insulin on dietary intake (Carvalheira et al., 2003; Posey et al., 2009; Morgan and Rahmouni, 2010). The RSNA responses to insulin may be affected by increased adiposity but this is still controversial and has only been investigated in mice (Rahmouni et al., 2003; Morgan and Rahmouni, 2010). The central effects of insulin in rats fed a HFD has not been examined to date.

Therefore, the main aims of the present study were to investigate the central effects of administration of resistin and insulin, both alone, and in combination, on RSNA in rats fed a normal diet chow, and whether HFD was able to influence the responses.

## Materials and Methods

### Ethics Statement

All the experimental protocols were performed in accordance with the Prevention of Cruelty to Animals Act 1986 (Australia). The procedures conform to the ‘Guiding Principles for Research Involving Animals and Human Beings,’ and the guidelines set out by the Australian Code of Practice for the Care and Use of Animals for Scientific Purposes, 2013 (National Health and Medical Research Council of Australia, Canberra, ACT, Australia) and were approved by the RMIT University Animal Ethics Committee (number 1347).

### Procedures

#### General

Male Sprague-Dawley rats (7–8 weeks of age) were obtained from Animal Resources Centre (Canning Vale, WA, Australia). Rats were acclimatized for 7 days after arrival and were housed at 23°C with a 12 h light–dark cycle. The rats were allowed free access to water and food; one group were fed with normal chow diet (ND) (4.8% fat), and the others were fed a high fat diet (HFD) (SF14-004 (22% fat), Speciality Feeds, Glen Forrest, WA, Australia) for 8–10 weeks. Body weight, food intake and caloric intake were monitored weekly. Caloric intake was calculated from the average food intake multiplied by the digestible energy (normal diet = 14 KJ/g, for High fat diet = 18 Kj/g).

An estimate of systolic blood pressure was made using a non-invasive tail cuff occlusion method of the caudal artery (NIBP in conjunction with a PowerLab system, ADInstruments, Sydney, NSW, Australia) and was performed fortnightly during the feeding period. For each time period, 3–4 measurements were made in each animal. The rats were acclimatized to the restraining device and procedures in the week prior to the commencement of the measurements. The percent whole body fat was determined at the end of the feeding period, using an EchoMRI 500 (Houston, TX, United States) in a sample of animals from each dietary treatment.

On the day of the experiments, rats were anesthetized using isoflurane gas (2–5% in O_2_). The femoral vein and artery were cannulated as previously described (Habeeballah et al., 2016b). The femoral vein was cannulated to maintain anesthesia using intravenous urethane (1.4–1.6 g/kg, supplemented with 0.05 ml of a 25% solution if required). The depth of anesthesia was maintained to ensure the absence of corneal and pedal reflexes. The femoral artery was cannulated for monitoring the arterial pressure. The mean arterial pressure (MAP) and heart rate (HR) were determined from the arterial pressure pulse using LabChart (ADInstruments, NSW, Australia). To maintain body temperature at approximately 37.5°C rats were kept on a heating pad during the experiments.

#### Microinjections Into the Lateral Brain Ventricle

Using a stereotaxic frame, rats were placed prone, and bregma and lambda were positioned on the same horizontal plane. A hole (2 mm diameter), centered 0.7 mm caudal to bregma and 1.4 mm lateral to the mid-line, was drilled into the skull, to expose the dorsal surface of the brain. Unilateral microinjections (5 μl) were made using a fine glass micropipette (50–70 μm tip diameter) inserted into the lateral brain ventricle (stereotaxic co-ordinates: 0.7 mm caudal to bregma, 1.4 mm lateral to the mid-line and 3.7–3.9 mm ventral to the surface of the dura). The micropipette was left in place for 1 min, after the microinjection. At the end of the experiment, a small amount of Pontamine Sky Blue was microinjected using the same coordinates to confirm microinjection into the lateral ventricle.

#### Renal Sympathetic Nerve Activity

After a flank incision, the left kidney was exposed retroperitoneally as previously described (Kosari et al., 2012; Habeeballah et al., 2016b). The renal nerve was isolated and dissected. The proximal end was placed onto electrodes and nerve activity was amplified using a low-noise differential amplifier (models ENG 187B and 133, Baker Institute, VIC, Australia), filtered (band pass 100–1,000 Hz), rectified, and integrated at 0.5 s intervals. The signal was recorded using a PowerLab data acquisition system (ADInstruments, NSW, Australia) and noise (determined after death of the animal) was subtracted from the recording as previously described (Habeeballah et al., 2016b).

#### Experimental Protocols

The MAP, HR, and RSNA were measured before and for 180 min after administration of saline (control) (*n* = 5 ND and HFD), resistin (7 μg; *n* = 4 ND, *n* = 5 HFD), insulin (500 mU; *n* = 6 ND, *n* = 5 HFD), and the combination of both resistin and insulin (*n* = 7 ND, *n* = 5 HFD; insulin was administered 15 min after resistin). The doses of each hormone were chosen on the basis of previous studies (Mark et al., 2009; Kosari et al., 2011; Habeeballah et al., 2016b). Only one injection per rat was made. At the end of the experiment, the epididymal fat was removed and weighed.

### Statistical Analysis

#### Within Diet

The average resting levels of MAP and HR before any intracerebroventricular (ICV) microinjections were compared between groups using one-way ANOVA. The integrated RSNA, averaged over 1–2 min, was calculated at 15-min intervals and expressed as a percentage of the resting level before the microinjections. Resting levels were determined over a 30 min period prior to ICV microinjection. Changes in MAP, HR, and RSNA over time following hormone/saline administration were compared between groups by using two-way ANOVA with repeated measures. When an overall ‘main effect’ significant difference between groups was detected, comparisons were made between individual groups, using Holm-Sidak’s test to compensate for multiple comparisons.

#### Comparisons Between High Fat and Normal Chow Diets

The changes in RSNA, MAP, and HR over time following resistin alone, insulin alone and the combination of insulin and resistin were compared between high fat and normal chow diets using two-way ANOVA with repeated measures. Data for resistin alone and saline (control) in ND and HFD has been reported by our lab previously (Habeeballah et al., 2016b, 2017) and are shown in this study for completeness of the comparisons. It should be noted that all studies were performed over a similar time frame. Furthermore, for the insulin alone, the data in the ND rats includes data previously published (Habeeballah et al., 2016b) but we have increased the amount of data by adding *n* = 2 more rats. All other data has not been published previously.

The body weight, average food intake, average caloric intake, and estimated systolic blood pressure during the feeding period were compared between dietary groups using two-way ANOVA. The percentage of whole body fat and the epididymal fat were compared between the dietary groups using Student’s unpaired *t*-test.

All results are expressed as means ± SEM. A value of *P* < 0.05 was considered to be statistically significant. PRISM software was used for statistical analysis and graphical representation (GraphPad, San Diego, CA, United States).

## Results

### Rats Fed a Normal Diet

#### Effect on RSNA

**FIGURE 1:**
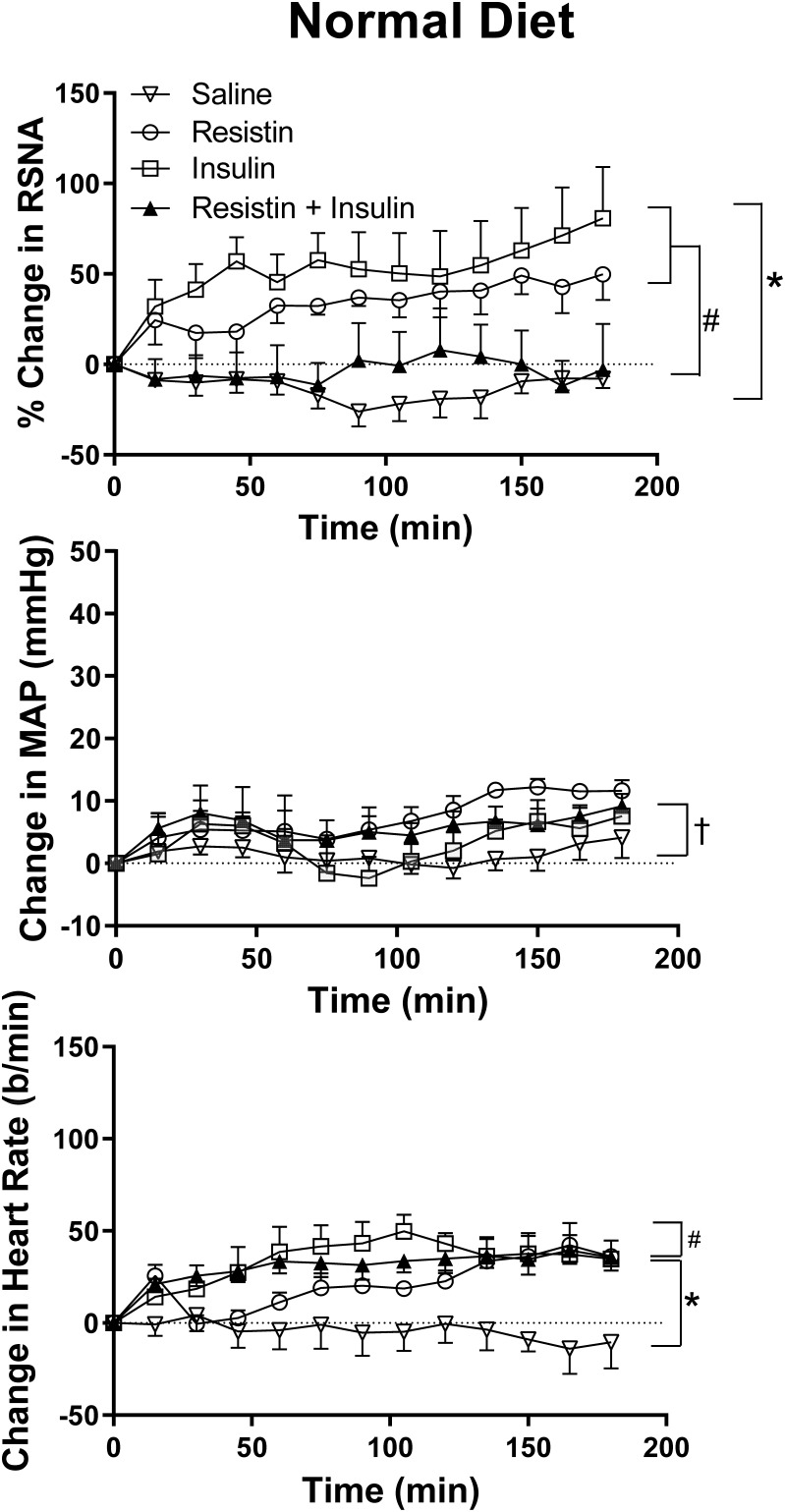
Changes in renal sympathetic nerve activity (RSNA) expressed as percent, mean arterial pressure (MAP), and heart rate (HR) over time induced by the intracerebroventricular administration of insulin (*n* = 6) (500 mU/5 μl), resistin (*n* = 4) (7 μg/5 μl), control (saline, *n* = 5) (5 μl), and the combination of resistin and insulin (*n* = 7) in rats fed a normal chow diet. Upper panel for RSNA ^∗^*P* < 0.0001 insulin vs. saline; ^#^*P* < 0.0001 insulin + resistin vs. insulin alone; and ^#^*P* < 0.0001 insulin + resistin vs. resistin alone. Middle panel for MAP ^†^*P* < 0.005 insulin vs. saline. Lower panel for HR ^∗^*P* < 0.0001 insulin + resistin vs. insulin alone and ^#^*P* < 0.0001 insulin + resistin vs. saline.

**FIGURE 2:**
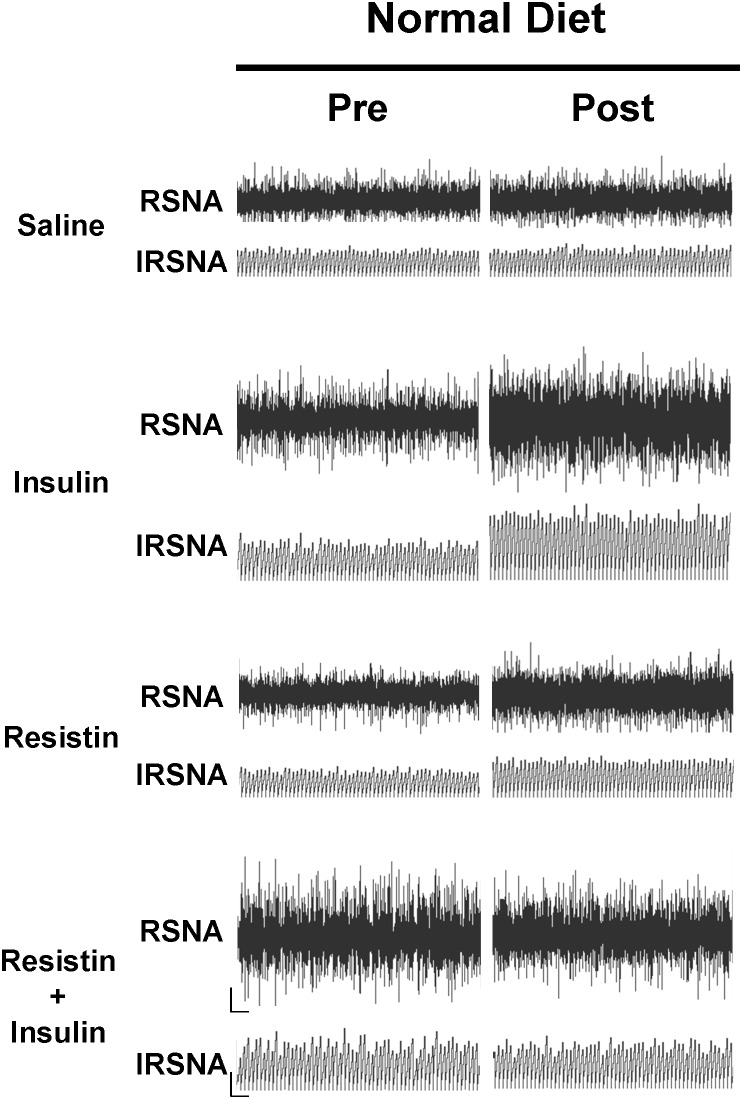
Examples of original tracings of renal sympathetic nerve activity (RSNA) and integrated renal sympathetic nerve activity (IRSNA) before and after the administration of saline (5 μl), insulin (500 mU/5 μl), resistin (7 μg/5 μl), and the combination of resistin and insulin in rats fed a normal diet. Horizontal bar = 2 s; vertical bar = 200 mV for RSNA and 10 mV.s for IRSNA.

In rats fed a normal diet, when insulin and resistin were given in combination, there was no increase in RSNA and this was not significantly different from the saline (control) group (Figure 1). However, this response was markedly different compared to the increases seen following insulin or resistin alone (*P* < 0.0001) (Figure 1). Insulin alone increased RSNA compared to saline (*P* < 0.0001) (Figure 1). Figure 2 shows examples of original tracings.

#### Effect on MAP and HR

There was an overall statistically significant difference between groups in the change in MAP (*P* < 0.0001). Following the administration of resistin and insulin in combination, there were no significant differences in the change in MAP compared to either hormone alone but there was a small difference from the saline (control) group (*P* < 0.005) (Figure 1). We do not consider this to be of physiological importance. ICV administration of insulin alone had no significant effect on MAP compared to saline (Figure 1).

There was also an overall difference in the HR responses between the groups (*P* < 0.0001). Subsequent analysis between individual groups showed that compared to saline, insulin alone and the combination of insulin with resistin increased HR (*P* < 0.0001) (Figure 1).

The resting levels of both MAP and HR before any ICV microinjection were not significantly different between groups (Table 1).

**Table 1:** Resting mean arterial pressure (MAP) and heart rate (HR) prior to intracerebroventricular injections in anesthetized rats fed normal chow (ND) or high fat diets (HFD).

	Saline		Insulin		Resistin		Insulin + Resistin	
	ND	HFD	ND	HFD	ND	HFD	ND	HFD
MAP (mmHg)	93.5±4.1	90.2±6.7	88.0±6.8	84.1±3.5	82.1±3.7	83.2±1.9	93.6±3.1	96.0±2.1
HR (b/min)	360±7	312±16	312±19	328±14	342±10	327±14	313±9	345±14


### Rats Fed a High Fat Diet (HFD)

#### Effect on RSNA

**FIGURE 3:**
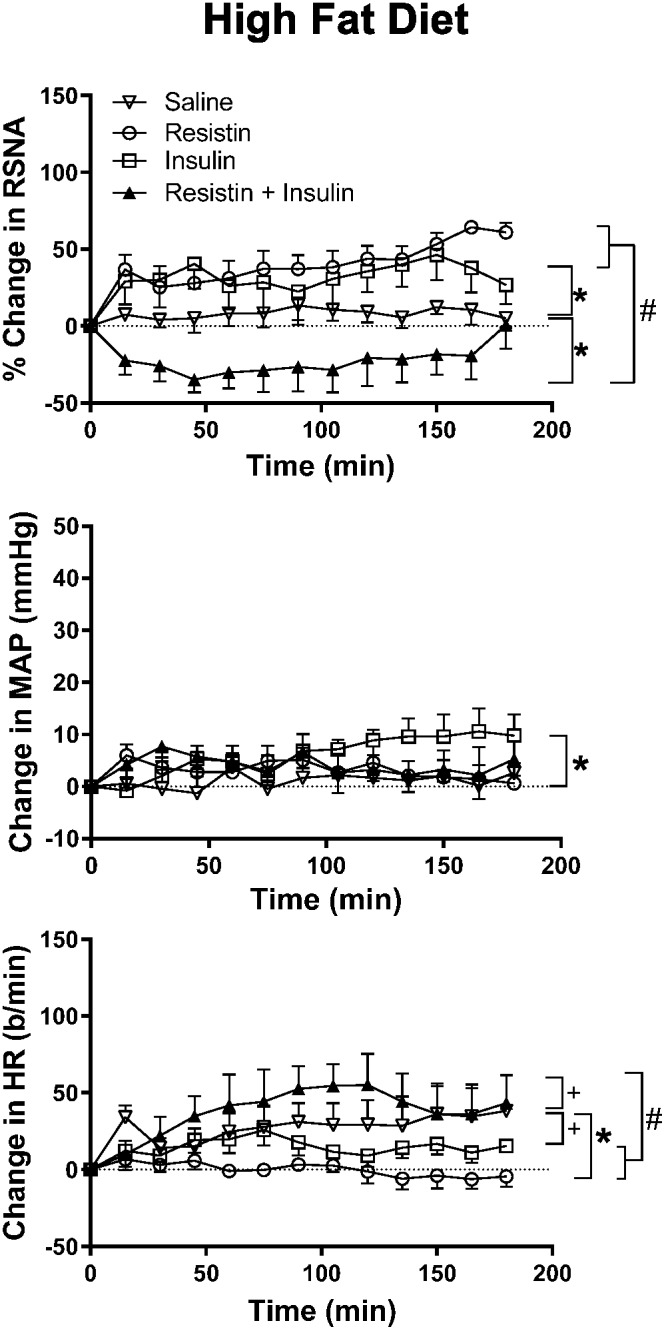
Changes in renal sympathetic nerve activity (RSNA) expressed as percent, mean arterial pressure (MAP), and heart rate (HR) over time induced by the intracerebroventricular administration of insulin (*n* = 5) (500 mU/5 μl), resistin (*n* = 5) (7 μg/5 μl), control (saline, *n* = 5) (5 μl) and the combination of resistin and insulin (*n* = 5) in rats fed a high fat diet. Upper panel, for RSNA ^∗^*P* < 0.0001 insulin vs. saline, ^∗^*P* < 0.0001 resistin + insulin vs. saline. ^#^*P* < 0.0001 resistin + insulin vs. insulin alone, *P* < 0.0001 resistin + insulin vs. resistin alone. Middle panel, for MAP ^∗^*P* < 0.005 insulin vs. saline. Lower panel, for HR ^+^*P* < 0.05 insulin vs. saline, ^+^*P* < 0.05 resistin + insulin vs. saline. ^#^*P* < 0.0001 resistin + insulin vs. insulin alone, ^#^*P* < 0.0001 resistin + insulin vs. resistin alone.

**FIGURE 4:**
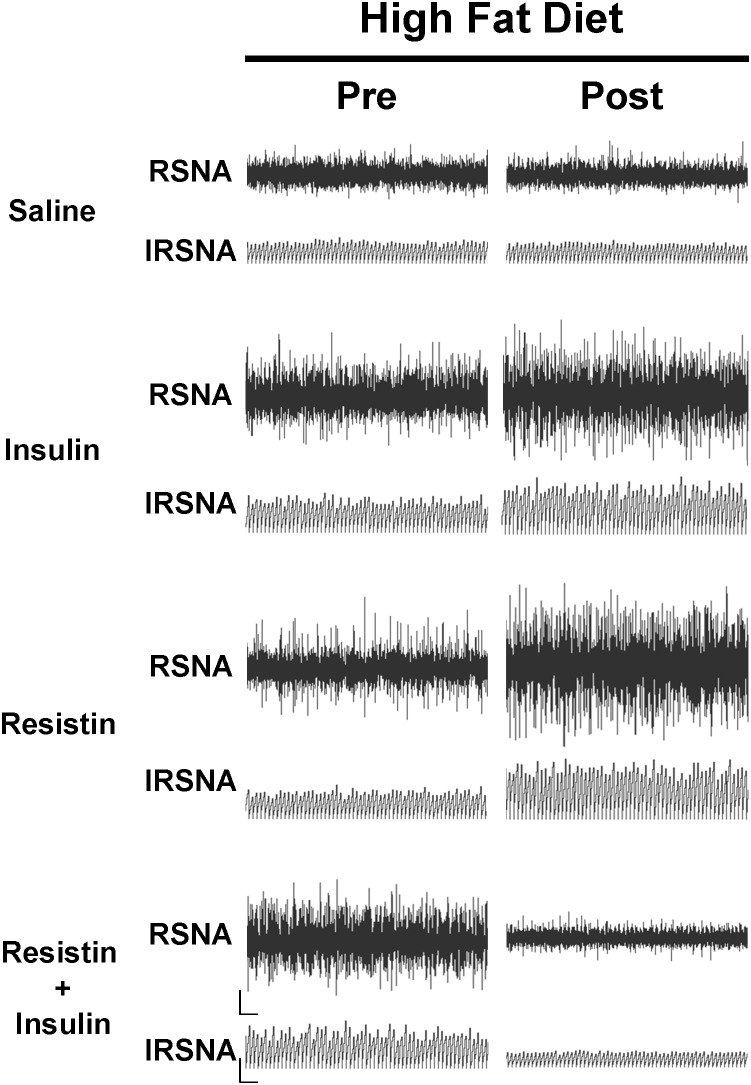
Examples of original tracings of renal sympathetic nerve activity (RSNA) and integrated renal sympathetic nerve activity (IRSNA) before and after the administration of saline (5 μl), insulin (500 mU/5 μl), resistin (7 μg/5 μl), and the combination of resistin and insulin in rats fed a high fat diet. Horizontal bar = 2 s; vertical bar = 200 mV for RSNA and 10 mV.s for IRSNA.

In rats fed a HFD, there was an increase in RSNA following ICV administration of insulin alone compared to saline (*P* < 0.0001) and reached a maximum of 46 ± 16% (Figure 3). By contrast, when resistin and insulin were administered in combination, RSNA fell by over 35% and was significantly different compared to saline (*P* < 0.0001) (Figure 3). The reduction in RSNA following the combined administration of resistin and insulin was significantly different from the increases observed following insulin alone (*P* < 0.0001) and resistin alone (*P* < 0.0001) (Figure 3). Examples of original tracings of RSNA pre and post each treatment in the rats fed a HFD are shown in Figure 4.

#### Effect on MAP and HR

There was an overall significant difference in the MAP response between groups (*P* < 0.01). The comparisons between individual groups showed that only the response to insulin was significantly different compared to saline (*P* < 0.005) (Figure 3).

In the HR response, there was also a significant difference overall. The comparison between individual groups showed that resistin alone (*P* < 0.0001), insulin alone (*P* < 0.05) and the combination (*P* < 0.05) were significantly different from saline (Figure 3).

When resistin and insulin were combined the HR response was significantly different from resistin alone and insulin alone (*P* < 0.0001) (Figure 3).

The resting levels of both MAP and HR prior to before any ICV microinjection did not show any significant differences between groups (Table 1).

### Comparison of Responses to Insulin and Resistin in Rats Fed High Fat vs. Normal Chow Diets

#### RSNA

**FIGURE 5:**
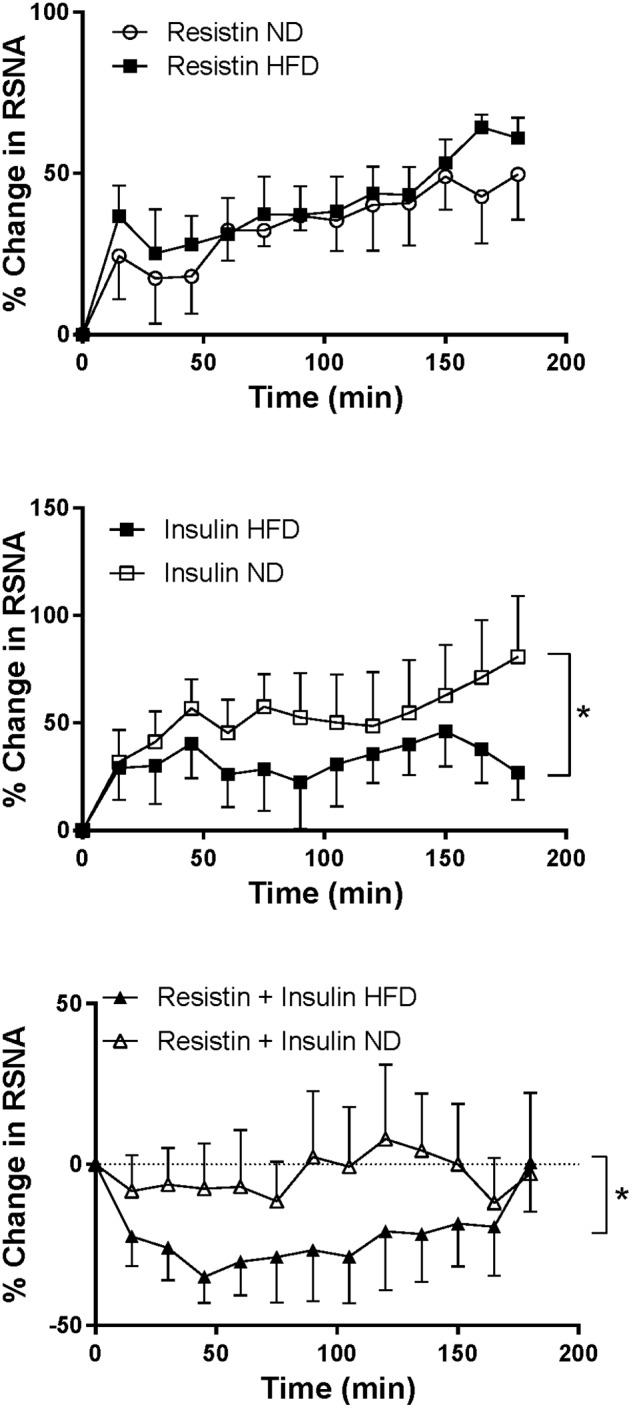
Renal sympathetic nerve activity (RSNA) responses over time between rats fed a normal chow (ND) and a high fat diet (HFD), elicited by intracerebroventricularly administered resistin (*n* = 4 ND, *n* = 5 HFD) (7 μg/5 μl), insulin (*n* = 6 ND, *n* = 5 HFD) (500 mU/5 μl) and the combination of resistin and insulin (*n* = 7 ND, *n* = 5 HFD). ^∗^*P* < 0.01 between groups.

Following combined insulin and resistin administration, there was a statistically significant difference in the RSNA response between the diets. In the HFD RSNA fell markedly compared to the ND (*P* < 0.01) (Figure 5).

In rats fed a HFD the increase in RSNA following insulin alone was significantly less than the increase seen in rats fed a ND (*P* < 0.01) (Figure 5). However, the response to resistin was not significantly different between dietary groups (Figure 5).

#### MAP and HR

**FIGURE 6:**
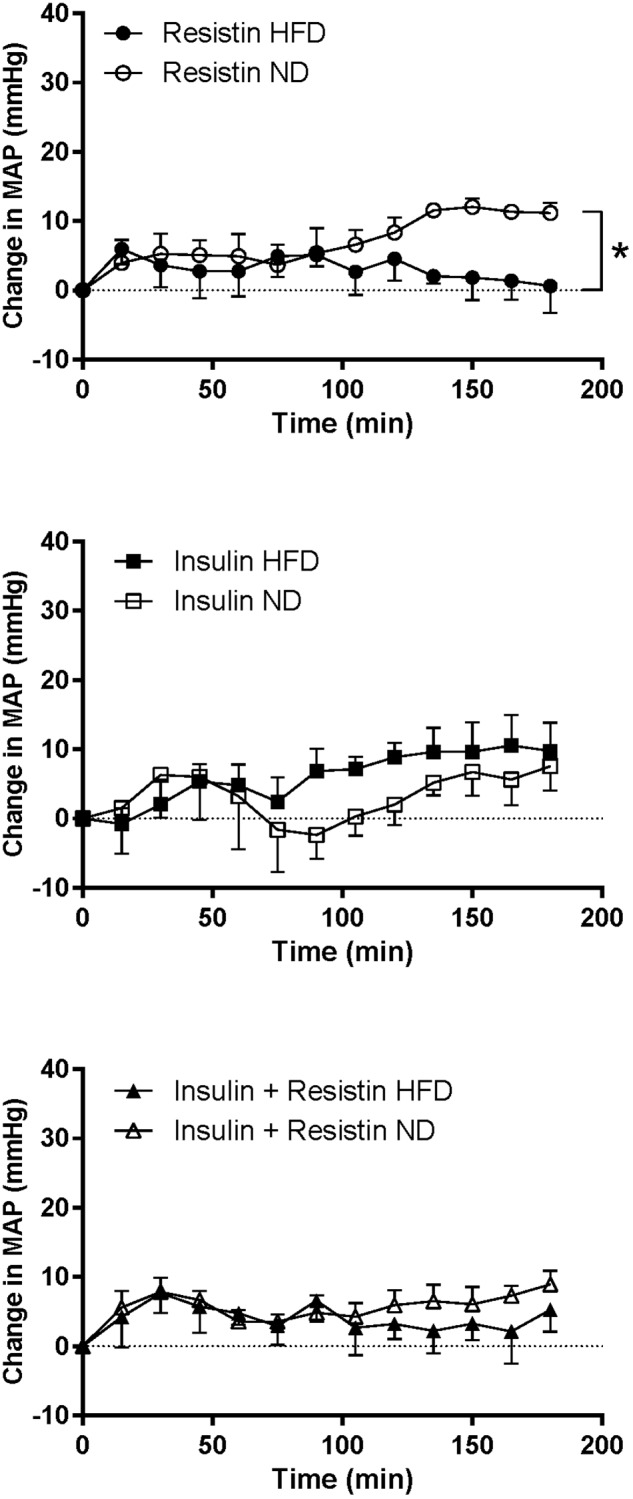
Mean arterial pressure (MAP) responses over time between rats fed a normal chow (ND) and a high fat diet (HFD), induced by intracerebroventricularly administered of resistin (*n* = 4 ND, *n* = 5 HFD) (7 μg/5 μl), insulin (*n* = 6 ND, *n* = 5 HFD) (500 mU/5 μl) and the combination of resistin and insulin (*n* = 7 ND, *n* = 5 HFD). ^∗^*P* < 0.001 between groups.

The response in MAP over time induced by insulin alone or in combination with resistin was not significantly different between the diets (Figure 6). However, the response to resistin was significantly less in rats fed a HFD compared to ND (*P* < 0.001) (Figure 6).

**FIGURE 7:**
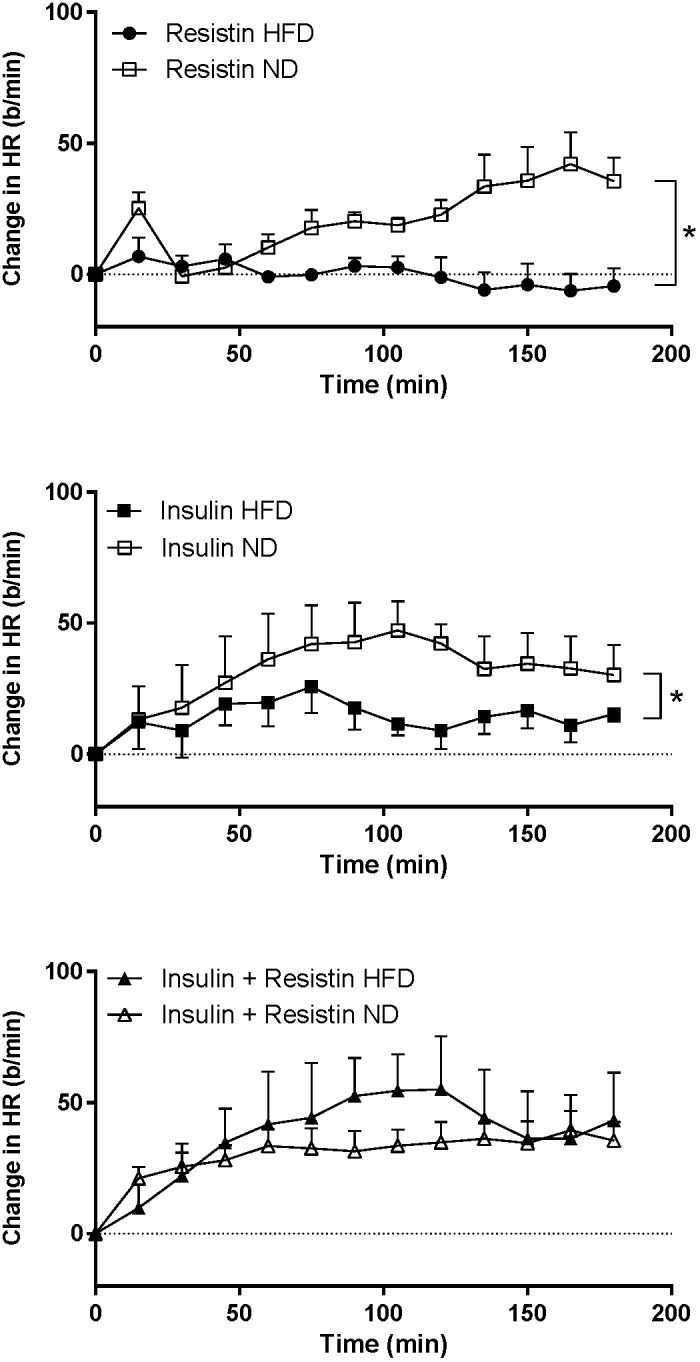
Heart rate (HR) responses over time between rats fed a normal chow (ND) and a high fat diet (HFD), induced by intracerebroventricularly administered of resistin (*n* = 4 ND, *n* = 5 HFD) (7 μg/5 μl), insulin (*n* = 6 ND, *n* = 5 HFD) (500 mU/5 μl) and the combination of resistin and insulin (*n* = 7 ND, *n* = 5 HFD). ^∗^*P* < 0.0001 between groups.

The HR response to insulin alone was significantly less in rats fed a HFD compared to rats fed a ND (*P* < 0.0001) (Figure 7). Similarly, resistin alone also induced a smaller HR response in the HFD group (*P* < 0.0001) (Figure 7). When resistin and insulin were administered in combination, the HR responses were not significantly different between the rats fed a HFD or ND (Figure 7). There were no significant differences in the resting basal MAP and HR before any ICV microinjection between diets (Table 1).

### Comparison of Systolic Blood Pressure and Metabolic Parameters in Rats Fed High Fat vs. Normal Chow Diets

#### Systolic Blood Pressure Changes During the Feeding Period

There was no significant difference observed in the systolic blood pressure over time between rats fed a HFD (105.5 ± 3.0, 109.2 ± 2.7 pre, post feeding, respectively) and rats fed a ND (106.0 ± 2.4, 112.3 ± 2.4 pre, post feeding, respectively).

### Metabolic Parameters

There was a significant increase in the percent of whole body fat mass in rats fed a HFD (13.8 ± 1.4%) compared to ND (8.0 ± 0.6%) (*P* < 0.005). Similarly, epididymal fat was increased significantly in the HFD (8.0 ± 0.6 g) compared to ND (6.1 ± 0.5 g) groups (*P* < 0.05).

### Body Weight, Food and Caloric Intake

**FIGURE 8:**
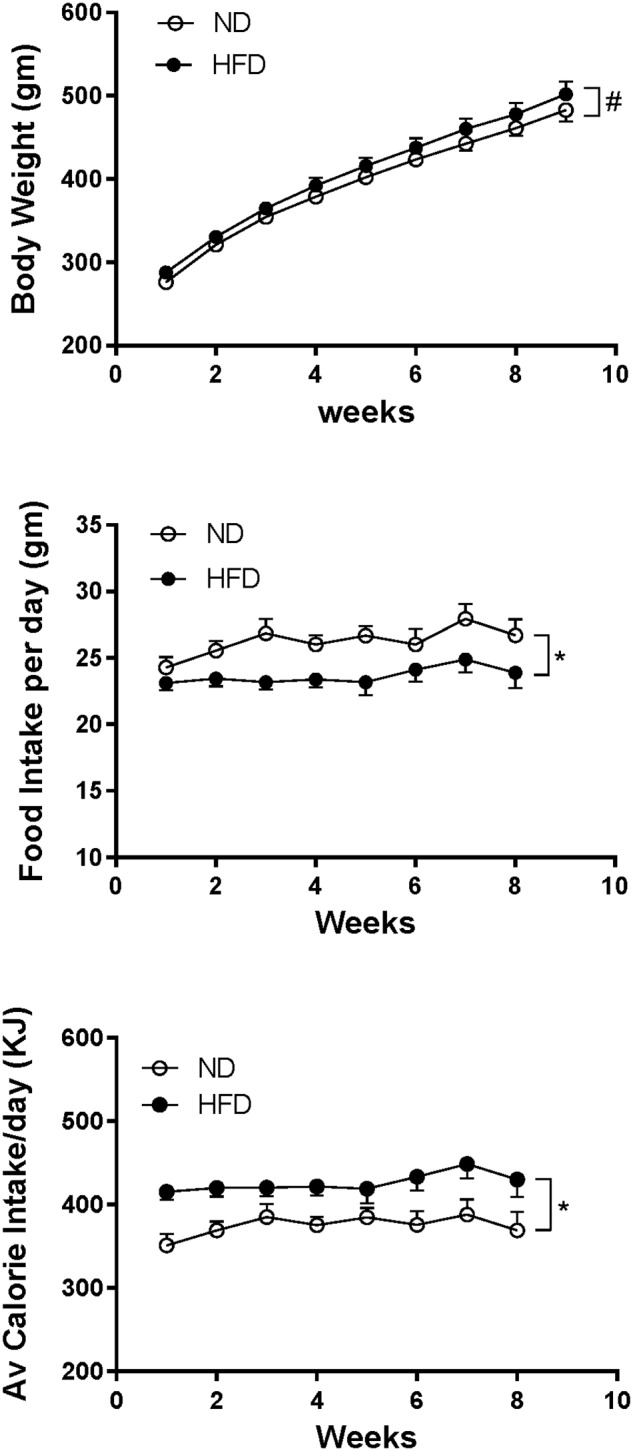
Effect of high fat diet (HFD; *n* = 18–19) on the body weight, food intake per day and average calorie intake per day over time compared to rats fed a normal chow diet (ND) (*n* = 18). ^#^*P* < 0.005 and ^∗^*P* < 0.0001 between groups.

In rats fed HFD, body weight increased over time and it was slightly but significantly greater compared to ND (*P* < 0.005) (Figure 8). Caloric intake was also greater in the HFD group (*P* < 0.0001), but the average daily food intake in the HFD fed rats was lower than in the ND group (*P* < 0.0001) (Figure 8).

## Discussion

The key findings of the present study are that the combination of resistin and insulin did not increase renal sympathetic nerve activity (RSNA) in rats fed a normal chow diet (ND) or the high fat diet (HFD). This contrasted with the sympatho-excitatory actions of each hormone alone. Additionally, in rats fed the HFD the increase in RSNA induced by insulin alone was smaller than in the group fed the normal chow diet.

We show for the first time that, in rats fed a HFD that insulin administered centrally increases RSNA to a lesser extent. than in rats fed a ND. This result is in agreement with reports in agouti obese mice (Morgan and Rahmouni, 2010). However, in another report in ob/ob obese mice the response to insulin was not less than in control lean mice (Rahmouni et al., 2003). Thus, the present study, would lend weight to the suggestion of Rahmouni et al. that HFD decreases the sensitivity to insulin.

The combination of resistin and insulin administered centrally did not increase RSNA in rats fed a normal chow diet or in the rats fed a high fat. This is in marked contrast to the clear sympatho-excitatory actions that each hormone alone induces in sympathetic nerve activity to the kidney. The current study suggests that there is an interaction in the brain pathways that mediate the responses in RSNA such that resistin may induce resistance to insulin’s renal sympatho-excitatory actions. This would be in accord with findings in the periphery where systemic administration of resistin induced resistance to the peripheral metabolic actions of insulin (Muse et al., 2004; Jiang et al., 2016; Santilli et al., 2016).

The mechanisms mediating the ability of resistin to induce resistance to the RSNA response elicited by insulin are unknown at present. However, the studies in peripheral tissues, including adipocytes, suggest several possible mechanisms. Resistin reduced the intracellular transduction pathway contributing to glucose homeostasis in adipocytes (Steppan et al., 2005). By reducing the phosphorylation of both the insulin receptor and of insulin receptor 1 substrate, and reducing activation of PI3-K and Akt and reducing production of phosphatidylinositol trisphosphate (Steppan et al., 2005). Resistin also increases the expression of suppressor of cytokine signaling 3 (SOCS3) which is known to reduce insulin signaling (Steppan et al., 2005).

The site(s) or nuclei within the central nervous system in which insulin and resistin may interact cannot be determined from injection made in the cerebral ventricles since the drug accesses the cerebrospinal fluid that bathes the brain. Insulin receptors are distributed widely in the central nervous system particularly the hypothalamus. In contrast, the receptors for resistin have not been clearly identified but sites in the brain which are likely sites of action also include the hypothalamus.

Resistin has been shown to induce resistance to insulin’s actions within the hypothalamus. Intracerebral ventricular infusion of resistin for 14 days into mice decreased insulin signaling by reducing the insulin-dependent phosphorylation of the insulin receptor, Akt and ERK 1/2 (Benomar et al., 2013). Interestingly, this was not seen in muscle, liver and adipose tissue (Benomar et al., 2013). These changes were associated with decreased insulin receptor expression and increased production of SOCS3 and phosphotyrosine phosphatase 1B (PTB-1B) (Benomar et al., 2013). Since resistin utilizes PI3-K whereas this enzyme is not involved in the RSNA response to insulin (Rahmouni et al., 2004; Kosari et al., 2012), it would suggest that a reduction in PI3-K is unlikely to account for the interaction in the RSNA effects we have observed. Thus, exploration of other mechanisms contributing to the interactions between resistin and insulin in the central nervous system regulation of RSNA will be an exciting focus for future studies.

Increased inflammatory processes also appear to be potential mechanisms that could contribute to increased insulin resistance. In the hypothalamus, resistin induced the activation of JNK and p38 MAPK and increased the expression of the pro-inflammatory cytokine IL-6 (Benomar et al., 2013). Additionally, resistin stimulated TLR-4 signaling pathways in the hypothalamus. This is interesting since these pathways lead to increases in inflammatory cytokines, and as such could provide a further mechanism through which resistin could induce insulin resistance (Benomar et al., 2013) in the brain Any inflammatory processes initiated by high fat feeding could further add to these mechanisms.

The lack of any increase in the RSNA following the administration of resistin and insulin described in the present study is in stark contrast to the response observed when resistin was combined with leptin that we have observed in earlier studies. In that work, the combination of resistin and leptin induced a greater increase in RSNA than that induced by either hormone alone. This was the case in rats fed a ND and in rats fed a HFD (Habeeballah et al., 2016b, 2017).

Whether the findings in the present study can be generalized to other sympathetic outflows is difficult to answer. It is worthy to note that both resistin and insulin increase lumbar sympathetic nerve activity, however, resistin decreases sympathetic nerve activity to brown adipose tissue which contrasts with the sympatho-excitatory effects on this outflow induced insulin (Morgan et al., 1993; Morgan and Rahmouni, 2010; Kosari et al., 2011; Habeeballah et al., 2016a,b, 2017). These data, together with the findings in the blood pressure and heart rate reponses in the present study (discussed further below), suggest that interactions between resistin and insulin may be selective for specific sympathetic outflows.

In the present study, systolic BP was not significantly elevated by the high fat diet. This is similar to previous studies using Wistar rats (Fardin et al., 2012). Sprague-Dawley rats may segregate into obese prone and obese resistant rats but only those prone to obesity are reported to have increased blood pressure (Dobrian et al., 2000; Stocker et al., 2007). Thus, elevations in blood pressure in rats fed high fat diets are variable and depend on various factors including the strain, amount of fat in the diet and the duration of feeding. In the present study we did not see any evidence of segregation or overt obesity. In contrast, rabbits appear to be very prone to obesity-induced hypertension since feeding for up to 4 weeks is sufficient to induce hypertension (Prior et al., 2010).

In our present work the body weights increased over time and the increase in rats fed the HFD was small. However, there was a clear redistribution of body fat. Thus, the interaction between insulin and resistin we observed in the rats fed the HFD occurs within the brain even in the absence of overt obesity, suggesting that high fat diets can influence the pathways mediating the changes in RSNA even in the absence of overt obesity.

## Summary

In overweight/obese conditions RSNA is abnormally elevated, and it contributes to the associated hypertension (Esler et al., 2006; Lim et al., 2013). Leptin makes a major contribution to the elevated RSNA in obesity associated hypertension (Lim et al., 2013) and all three hormones can be elevated. In a previous study, in rats fed a HFD, we found that centrally administered resistin combined with leptin resulted in a greater increase in RSNA than that produced by each hormone alone (Habeeballah et al., 2017). The present results indicate that this is not the case for resistin combined with insulin. Although very speculative, we suggest that the sympatho-excitatory effects of resistin in combination with leptin may be the predominant interaction.

## Author Contributions

The work was performed in the laboratory of EB. EB devised the study, analyzed the data, interpreted the data, and wrote manuscript. HH and NA performed the experiments, analyzed and interpreted the data, and contributed to writing the manuscript. MS contributed to the manuscript and interpretation of the data. All authors approved the final version of the manuscript and agree to be accountable for all aspects of the work in ensuring that questions related to the accuracy or integrity of any part of the work are appropriately investigated and resolved. All persons designated as authors qualify for authorship, and all those who qualify for authorship are listed.

## Conflict of Interest Statement

The authors declare that the research was conducted in the absence of any commercial or financial relationships that could be construed as a potential conflict of interest.
